# Sex hormone receptor expression and survival in esophageal adenocarcinoma: a prospective cohort study

**DOI:** 10.18632/oncotarget.26236

**Published:** 2018-10-19

**Authors:** Úna C. McMenamin, James Trainor, Helen G. Coleman, Damian T. McManus, Stephen McQuaid, Victoria Bingham, Jacqueline James, Manuel Salto-Tellez, Brian T. Johnston, Richard C. Turkington

**Affiliations:** ^1^ Cancer Epidemiology Research Group, Centre for Public Health, Queen's University Belfast, Belfast, Northern Ireland, UK; ^2^ Department of Pathology, Belfast Health and Social Care Trust, Belfast, Northern Ireland, UK; ^3^ Northern Ireland Molecular Pathology Laboratory, Centre for Cancer Research and Cell Biology, Queen's University Belfast, Belfast, Northern Ireland, UK; ^4^ Department of Gastroenterology, Royal Victoria Hospital, Belfast Health and Social Care Trust, Belfast, Northern Ireland, UK; ^5^ Centre for Cancer Research and Cell Biology, Queen's University Belfast, Belfast, Northern Ireland, UK

**Keywords:** estrogen receptor, androgen receptor, esophageal adenocarcinoma, survival, recurrence

## Abstract

**Introduction:**

A striking epidemiological feature of esophageal adenocarcinoma (EAC) is its strong, unexplained male predominance but few studies have evaluated the prevalence of sex hormone receptor expression in EAC.

**Results:**

A low proportion of EAC tumors stained positive for ERα (4%) and AR (3%) while approximately one third stained positive for ERβ (31%). After a mean follow-up of 3 years (max 9 years), no significant associations were seen for ERα, ERβ or AR expression and EAC recurrence or survival. A non-significant reduction in mortality was observed for positive ERβ tumor expression, when restricting to patients with gastro-esophageal junctional (GEJ) cancer (HR 0.58, 95% CI 0.33, 1.03, *p* = 0.06).

**Materials and Methods:**

We identified all EAC patients who underwent neo-adjuvant chemotherapy prior to surgical resection between 2004–2012 in the Northern Ireland Cancer Centre. Immunohistochemical expression of ERα, ERβ and AR was scored on triplicate cores to generate H-scores. Cox proportional hazards regression was used to evaluate the association between sex hormone receptor expression and overall, cancer-specific and recurrence-free survival.

**Conclusion:**

We found little evidence of ERα or AR expression in EAC. A moderate proportion expressed ERβ and there was suggestive evidence that its expression was associated with improved survival in GEJ cancer patients.

## INTRODUCTION

The incidence of esophageal adenocarcinoma (EAC) is rising and prognosis remains poor [1, 2]. Despite advances in oncological and surgical management the five-year survival rate for EAC is only 15%, and even in early localized disease treated with peri-operative chemotherapy, survival is less than 40% [3, 4]. Attempts to improve prognosis have focused on the surveillance of Barrett's esophagus, the precursor to EAC, but only a small proportion of patients have a previous Barrett's diagnosis [5, 6]. The most striking epidemiological feature of EAC is its strong unexplained male predominance [7–9], with male-to-female incidence ratios of up to 6:1 observed [10, 11]. This has led to the suggestion of sex hormone involvement in EAC development, possibly via estrogenic protection, a detrimental effect of androgens, or both [12, 13].

Recent epidemiological evidence demonstrates associations between circulating sex steroid hormones and EAC risk [14]. Moreover, prognosis after surgical resection of EAC has been shown to be better in females compared to males [15, 16]. Mounting preclinical evidence supports the hypothesis that sex hormones may be important in EAC pathogenesis [7]. Estrogens decrease tumor growth and increase apoptosis in EAC cell lines [17], while estrogen replacement has been shown to suppress esophageal damage of reflux esophagitis in animal models [18] and reduce esophageal tumor growth [19]. Androgen receptor (AR) is a key mediator of inflammatory signals in esophageal cancer progression and its expression has been shown to promote cell migration, invasion and proliferation in esophageal cancer *in vivo* [13, 20].

Although the exact mechanisms underlying sex hormone involvement in esophageal carcinogenesis remain unclear, it is potentially mediated through sex hormone receptors including estrogen receptors (ER) and AR [21]. ERα is predominantly expressed in female sex organs including the breast, uterus and ovaries while ERβ is widely expressed in many other tissues, including the esophagus, in both males and females [13, 22]. Other sex hormone receptors include the progesterone receptor, activated by the steroid hormone progesterone, and related receptors include G protein-coupled estrogen receptors (GPER), such as G protein-coupled estrogen receptor 1 or GPR30, which exert their physiological effects through ERα and β via pregenomic pathways [23]. Clinical studies have shown complex patterns of ER expression in EAC specifically but few studies have been conducted and to date they have been small in size (e.g. 11–28 cases) [24–28]. Similarly, few studies have investigated AR expression in EAC tissue [27, 29, 30]. The association between sex hormone receptor expression and EAC prognosis has also received little attention; one study reported an inverse association between ERβ expression and cancer-specific survival [24] while another observed no association between AR expression and overall survival [30].

Considering that ER and AR signalling can be modulated through existing therapeutic selective targeting (in addition to emerging novel techniques such as selective ERβ agonists), further understanding of the role of sex hormone receptors in EAC is important considering that molecular-targeted intervention may offer opportunities for primary and secondary prevention. In a UK cohort study, we aimed to determine prevalence of ERα, ERβ and AR expression in EAC tissue, and to investigate the influence of sex hormone receptor expression on EAC recurrence and survival.

## RESULTS

### Patient cohort

**Figure 1:**
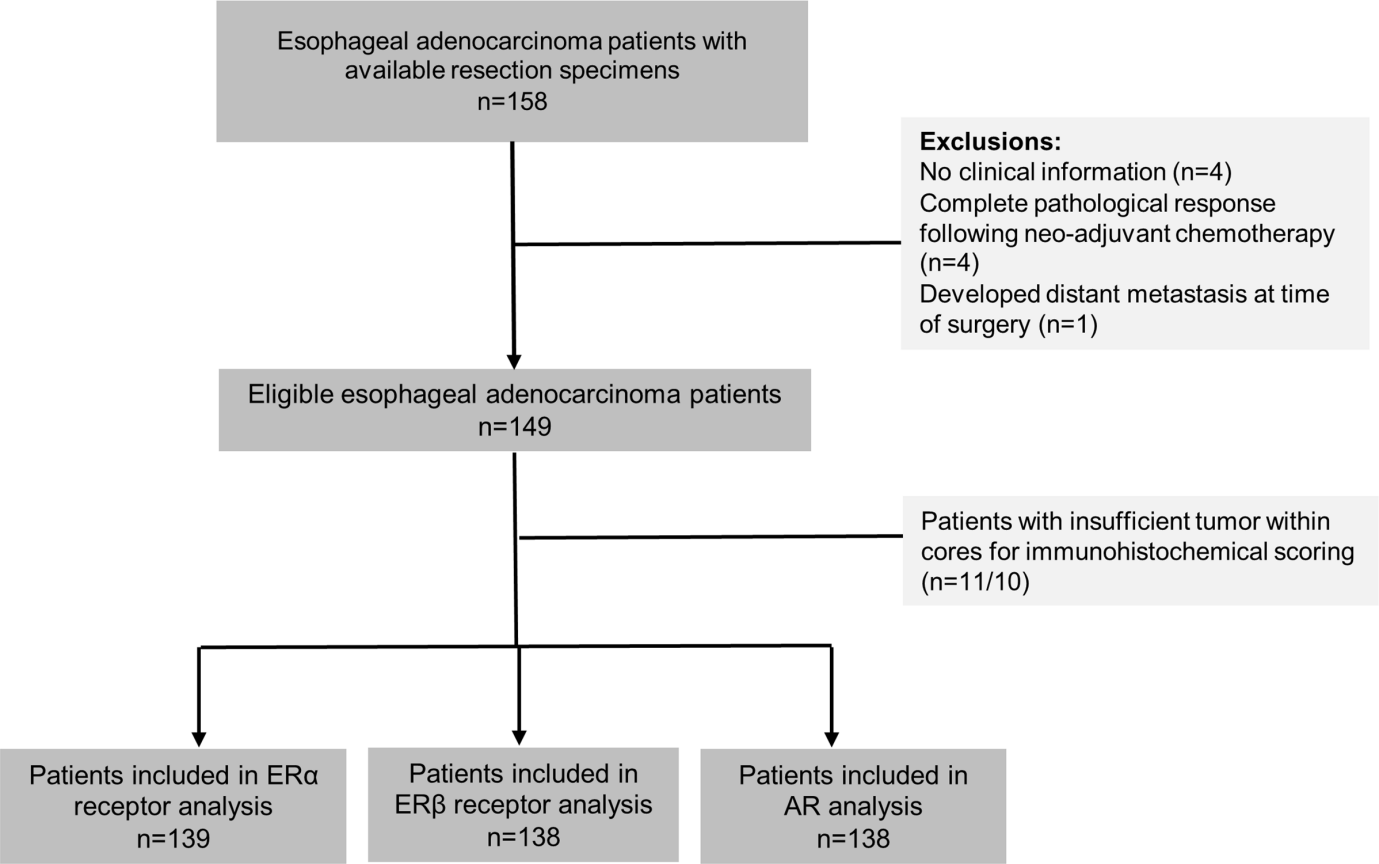
Study selection process for analysis of ERα, ERβ and AR expression in esophageal adenocarcinoma patients Abbreviations: ER = estrogen receptor, AR = androgen receptor, α = alpha, β = beta.

During the study period, a total of 158 formalin-fixed paraffin embedded (FFPE) EAC resection specimens were collected from the Northern Ireland Cancer Centre. Clinical information was available for 154 of these patients. Four patients were excluded on the basis of complete pathological response (ypT0) following neo-adjuvant chemotherapy, in addition to one patient who had metastases present at the time of surgery. Patients were additionally excluded if they lacked sufficient tumor within cores for immunohistochemical scoring leaving a total of 139, 138 and 138 in analysis of ERα, ERβ and AR, respectively, see Figure 1. The majority of included patients were male (78%) and average age at EAC diagnosis was 63 years. Most patients had tumors located in the gastro-esophageal junctional (GEJ) (83%).

**Figure 2:**
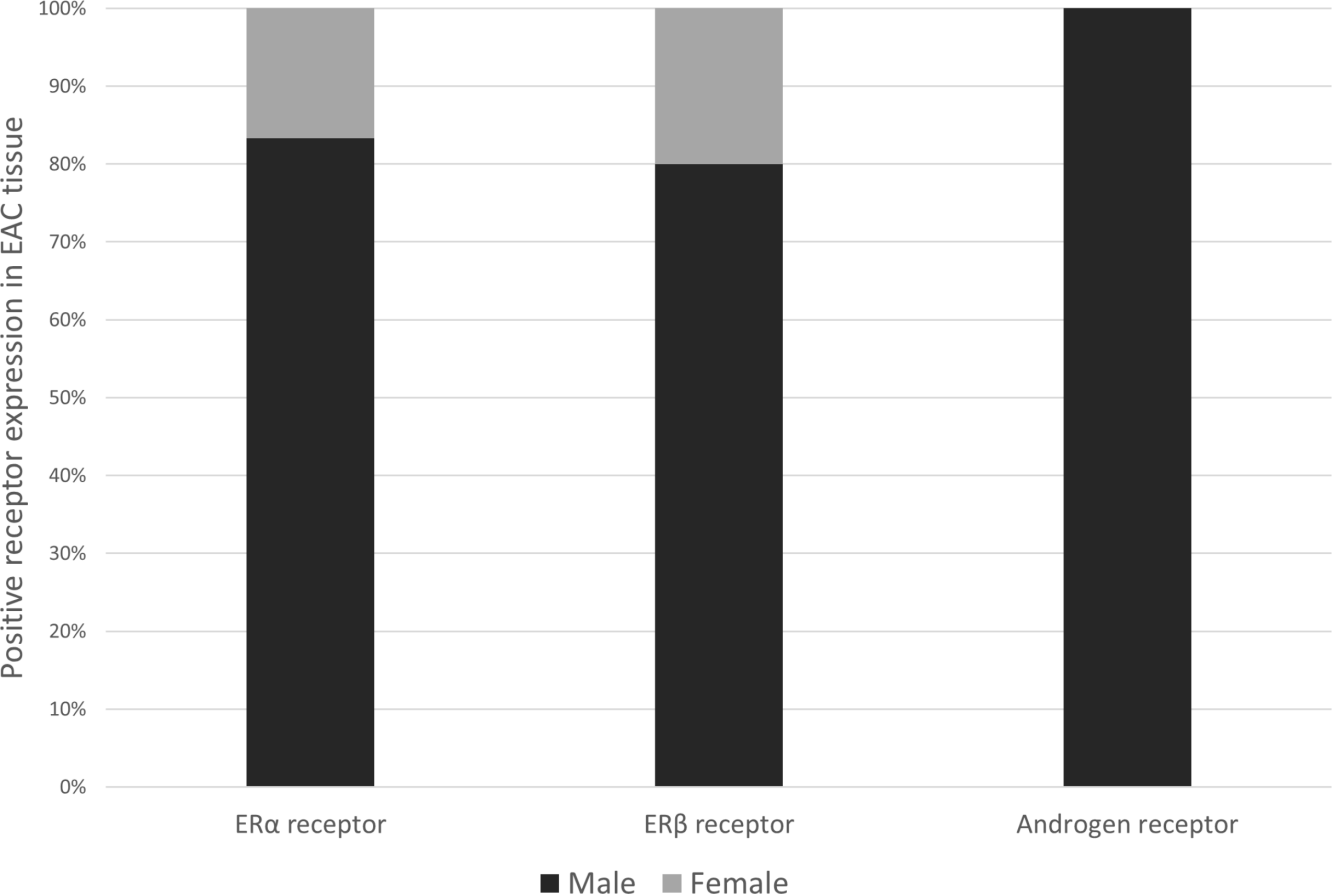
Bar chart showing sex distribution (%) among esophageal adenocarcinoma patients who scored positively for ERα (*n* = 6), ERβ (*n* = 43) or AR (*n* = 4) expression Abbreviations: ER = estrogen receptor, AR = androgen receptor, α = alpha, β = beta.

Patient characteristics by sex hormone receptor status are listed in Table 1. Although the prevalence of positive sex hormone receptor expression did not significantly differ by sex, the majority of ERα and ERβ positive expression was in male EAC patients (83.3% and 79.1%, respectively), while positive expression of AR was only observed in males, see Figure 2.

**Table 1:** Characteristics of EAC patients by sex hormone receptor expression

	ERα receptor status			ERβ receptor status			AR status		
	Negative (*n* = 133)	Positive (*n* = 6)	*P*	Negative (n = 95)	Positive (*n* = 43)	*P*	Negative (*n* = 134)	Positive (*n* = 4)	*P*
**Sex**									
Male	104 (78.2)	5 (83.3)	0.76	74 (77.9)	34 (79.1)	0.88	104 (77.6)	4 (100)	0.28
Female	29 (21.8)	1 (16.7)		21 (22.1)	9 (20.9)		30 (22.4)	0	
**Age at diagnosis (years)**									
<50	13 (9.8)	1 (16.7)	0.71	10 (10.5)	4 (9.3)	0.94	14 (10.4)	0	0.60
50–59	24 (18.0)	2 (33.3)		18 (18.9)	10 (23.3)		26 (19.4)	0	
60–70	63 (47.4)	2 (33.3)		45 (47.4)	19 (44.2)		62 (46.3)	3 (75)	
≥70	33 (24.8)	1 (16.7)		22 (23.2)	10 (23.3)		32 (23.9)	1 (25)	
**Smoking status**									
Non-smoker	34 (29.1)	1 (16.7)	0.38	24 (28.2)	12 (32.4)	0.52	34 (25.4)	1 (25)	0.38
Ex-smoker	29 (24.8)	3 (50)		20 (23.5)	12 (32.4)		32 (23.9)	0	
Current smoker	54 (46.1)	2 (33.3)		41 (48.2)	13 (35.1)		52 (38.8)	3 (75)	
*Unknown*	*16*	*0*		*10*	*6*		*16*	*0*	
**Alcohol**									
Non-drinker	42 (37.5)	2 (33.3)	0.84	28 (34.6)	16 (44.4)	0.58	42 (37.2)	1 (25)	0.62
Drinker	70 (62.5)	4 (66.7)		53 (65.4)	20 (55.6)		71 (62.8)	3 (75)	
*Unknown*	*21*	*0*		*14*	*7*		*21*	*0*	
**Primary tumor site**									
Lower third	22 (16.5)	0	0.28	15 (15.8)	7 (16.3)	0.94	22 (16.4)	0	0.38
Gastro-esophageal junction	111 (83.5)	6 (100)		80 (84.2)	36 (83.7)		112 (83.6)	4 (100)	
**Siewert classification^a^**									
Siewert I	65 (58.6)	4 (66.7)	0.69	48 (60)	21 (58.3)	0.87	66 (58.9)	2 (50)	0.72
Siewert II/III	46 (41.4)	2 (33.3)		32 (40)	15 (41.7)		46 (41.1)	2 (50)	
**PET response**									
No	40 (40.8)	4 (66.7)	0.21	28 (39.4)	16 (48.5)	0.38	44 (43.6)	0	0.22
Yes	58 (59.2)	2 (33.3)		43 (60.6)	17 (51.5)		57 (56.4)	2 (100)	
*Unknown*	*35*	*0*		*24*	*10*		*33*	*2*	
**Lymphatic vascular invasion**									
No	40 (30.3)	3 (50)	0.31	29 (30.8)	13 (30.2)	0.94	42 (31.6)	0	0.39
Yes	92 (69.7)	3 (50)		65 (69.2)	30 (69.8)		91 (68.4)	4 (100)	
*Unknown*	*1*	*0*		*1*	*0*		*1*	*0*	
**Grade**									
Well	4 (3)	0	0.87	4 (4.2)	0	0.32	4 (3)	0	0.84
Moderate	51 (38.3)	2 (33.3)		37 (38.9)	15 (34.9)		50 (37.3)	2 (50)	
Poor	78 (58.7)	4 (66.7)		54 (56.8)	28 (65.1)		80 (59.7)	2 (50)	
**Circumferential resection margin status**									
Negative	72 (54.5)	3 (50)	0.83	54 (57.5)	19 (44.2)	0.15	73 (54.9)	1 (25)	0.24
Positive	60 (45.5)	3 (50)		40 (42.5)	24 (55.8)		60 (45.1)	3 (75)	
*Unknown*	*1*	*0*		*1*	*0*		*1*	*0*	
**Surgical T stage**									
1	11 (8.3)	1 (16.7)	0.87	8 (8.4)	4 (9.3)	0.95	11 (8.2)	1 (25)	0.53
2	26 (19.6)	1 (16.7)		16 (16.8)	8 (18.6)		26 (19.4)	0	
3	91 (68.4)	4 (66.7)		67 (70.5)	30 (69.8)		92 (68.7)	3 (75)	
4	5 (3.8)	0		4 (4.2)	1 (2.3)		5 (3.7)	0	
**Surgical N stage**									
0	45 (33.8)	2 (33.3)	0.04	31 (32.6)	15 (34.8)	0.23	45 (33.6)	1 (25)	0.51
1	29 (21.8)	0		23 (24.2)	6 (14)		28 (20.9)	1 (25)	
2	32 (24.1)	0		23 (24.2)	8 (18.6)		30 (22.4)	2 (50)	
3	27 (20.3)	4 (66.7)		18 (19)	14 (32.6)		31 (23.1)	0	

^a^Restricted to patients with gastro-esophageal junction tumors; Siewert classification 1: adenocarcinoma of the distal esophagus, 2: true carcinoma of the cardia arising at the esophago-gastric junction, 3: subcardial gastric cancer infiltrating distal esophagus. ER = estrogen receptor, α = alpha, β = beta, AR = androgen receptor, GEJ = gastro-esophageal junction, PET = positron emission tomography, T = tumor, N = nodal.

Although only a minority of tumors expressed ERα, patients whose tumors expressed ERα were more likely to have three or more disease positive nodes (i.e. at least ypN2 disease) compared to patients with negative expression. Demographic and lifestyle factors did not differ significantly according to sex hormone receptor status and similarly, there were no significant differences in tumor characteristics by ERα, ERβ or AR expression (Table 1). Average duration of follow-up was 3 years and ranged from 4 months to 9 years.

### Sex hormone receptor expression

**Figure 3:**
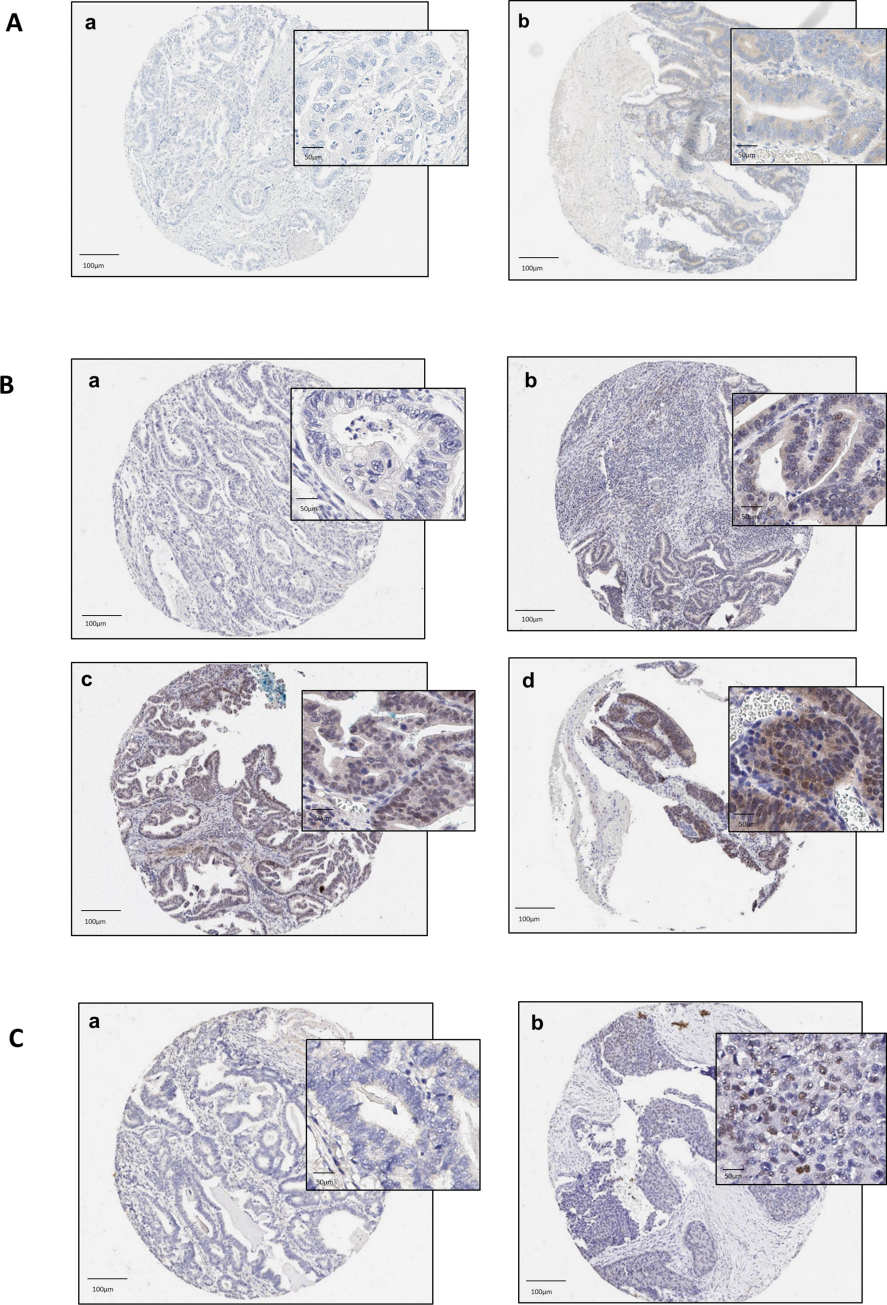
(**A**–**C**) Tissue microarray staining in esophageal adenocarcinoma core samples according to ERα (A), ERβ (B) and AR expression. a: no staining [intensity 0]; b: weak staining [intensity 1]; c: moderate staining [intensity 2]; d: strong staining [intensity 3]. NB. No cores had ER-alpha and androgen receptor biomarkers scored at intensity 2/3. Abbreviations: ER = estrogen receptor, AR = androgen receptor, α = alpha, β = beta.

Figure 3A–3C shows the immunohistochemical cellular staining according to positive and negative ERα expression. A low proportion of positive (nuclear) ERα staining was observed in 6 out of 139 patients (4.3%), Figure 3A. A moderate proportion of EAC tumors were found to positively express ERβ, which was predominantly nuclear in nature (43 out of 138 patients (31%)) (Figure 3B). A small proportion of EAC tumors were positive for AR nuclear expression (4 out of 138 (2.9%)) (Figure 3C). Overall, the observed staining was largely homogenous in nature.

### ERα expression and EAC progression

During follow-up, 86 cancer recurrences, 78 EAC-specific deaths and 83 deaths from any cause occurred. Results for ERα expression and associated risk of EAC clinical outcomes are listed in Table 2. Unadjusted analysis did not demonstrate any significant associations between ERα expression and risk of cancer recurrence, cancer-specific or overall survival and results were similar following adjustment for potential confounders (HR 1.32, 95% CI 0.41, 4.24), (HR 1.14, 95% CI 0.29, 4.50) and (HR 1.36, 95% CI 0.44, 4.23), respectively.

**Table 2:** Overall survival, cancer-specific survival and recurrence-free survival according to sex hormone receptor expression

	Overall survival					Cancer-specific survival					Recurrence-free survival			
Biomarker	Events	Patients	Unadjusted HR (95% CI)	Adjusted HR^a^ (95% CI)		Events	Patients	Unadjusted HR (95% CI)	Adjusted HR^a^ (95% CI)		Events	Patients	Unadjusted HR (95% CI)	Adjusted HR^a^ (95% CI)
*Estrogen receptor α*														
Negative	79	133	1.00	1.00		75	129	1.00	1.00		82	129	1.00	1.00
Positive	4	6	1.45 (0.53, 3.98)	1.32 (0.41, 4.24)		3	5	1.21 (0.38, 3.87)	1.14 (0.29, 4.50)		4	6	1.29 (0.17, 2.79)	1.36 (0.44, 4.23)
*Estrogen receptor β*														
Negative	56	95	1.00	1.00		52	91	1.00	1.00		56	91	1.00	1.00
Positive	28	43	1.14 (0.72, 1.80)	0.71 (0.41, 1.21)		27	42	1.14 (0.72, 1.82)	0.73 (0.42, 1.28)		31	43	1.28 (0.82, 2.00)	0.89 (0.53, 1.48)
*Androgen receptor*														
Negative	79	134	1.00	1.00		74	129	1.00	1.00		82	130	1.00	1.00
Positive	4	4	2.29 (0.83, 6.33)	1.50 (0.49, 4.62)		4	4	2.42 (0.88, 6.72)	1.48 (0.48, 4.55)		4	4	1.98 (0.72, 5.43)	1.50 (0.49, 4.57)

Abbreviations: HR = Hazard ratio, CI = Confidence interval, α = alpha, β = beta.

^a^Adjusted for age at diagnosis, sex, nodal status, grade, PET response, circumferential resection margin status, lymphatic vascular invasion, primary site, smoking.

### ERβ expression and EAC progression

In analysis of ERβ expression, a total of 87 recurrences, 79 cancer-specific deaths and 84 deaths from any cause were observed during follow-up. In adjusted analyses, improvements in overall survival (HR 0.71, 95% CI 0.41, 1.21), cancer-specific survival (HR 0.73, 95% CI 0.42, 1.28) and recurrence-free survival (HR 0.89, 95% CI 0.53, 1.48) were observed for patients whose tumors expressed ERβ compared to those whose tumors were negative for ERβ, however results were not statistically significant.

### AR expression and EAC progression

In analysis of AR expression, there were 86 cancer recurrences, 78 deaths from EAC and 83 deaths from any cause identified during follow-up. No significant associations were observed for AR positive expression and risk of EAC outcomes, Table 2. Although HRs were raised in unadjusted analysis, they attenuated following adjustment for potential confounders and remained non-significant; overall survival (adjusted HR 1.50, 95% CI 0.49, 4.62), cancer-specific survival (HR 1.48, 95% CI 0.48, 4.55) and recurrence-free survival (HR 1.50, 95% CI 0.49, 4.57).

### Sensitivity analysis

Table 3 presents the results of sensitivity analyses restricting the cohort to patients diagnosed with tumors located in the GEJ. Overall, results were similar to the main analysis with no significant associations observed for ERα or AR expression and risk of any outcome following adjustment for potential confounders. ERβ expression was associated with a reduced risk of death from any cause (HR 0.58, 95% CI 0.33, 1.03, *p* = 0.06) and reduced cancer-specific mortality (HR 0.59, 95% CI 0.32, 1.07, *p* = 0.08), although results failed to reach statistical significance. A less marked (non-significant) association was seen for ERβ expression and recurrence-free survival (HR 0.75, 95% CI 0.43, 1.29).

**Table 3:** Sex hormone receptor expression and EAC progression restricting to patients with gastro-esophageal junction tumors

	Overall survival					Cancer-specific survival					Recurrence-free survival			
Biomarker	Events	Patients	Unadjusted HR (95% CI)	Adjusted HR^a^ (95% CI)		Events	Patients	Unadjusted HR (95% CI)	Adjusted HR^a^ (95% CI)		Events	Patients	Unadjusted HR (95% CI)	Adjusted HRa (95% CI)
*Estrogen receptor α*														
Negative	69	111	1.00	1.00		65	107	1.00	1.00		71	108	1.00	1.00
Positive	4	6	1.40 (0.51, 3.87)	1.18 (0.36, 3.90)		3	5	1.18 (0.37, 3.78)	1.00 (0.24, 4.08)		4	6	1.16 (0.42, 3.18)	1.20 (0.38, 3.77)
*Estrogen receptor β*														
Negative	50	80	1.00	1.00		46	76	1.00	1.00		46	77	1.00	1.00
Positive	24	36	1.00 (0.61, 1.64)	0.58 (0.33, 1.03)		23	35	0.99 (0.60, 1.64)	0.59 (0.32, 1.07)		23	36	1.14 (0.71, 1.84)	0.75 (0.43, 1.29)
*Androgen receptor*														
Negative	69	112	1.00	1.00		64	107	1.00	1.00		71	109	1.00	1.00
Positive	4	4	2.22 (0.80, 6.18)	1.77 (0.55, 5.67)		4	4	2.38 (0.85, 6.63)	1.70 (0.53, 5.47)		4	4	1.91 (0.69, 5.26)	2.07 (0.66, 6.51)

Abbreviations: HR = Hazard ratio, CI = Confidence interval, α = alpha, β = beta.

^a^Adjusted for age at diagnosis, sex, nodal status, grade, PET response, circumferential resection margin status, lymphatic vascular invasion, smoking.

## DISCUSSION

In a population-representative study of EAC patients who underwent neoadjuvant chemotherapy prior to surgical resection, we found little evidence of ERα or AR expression in EAC while expression of ERβ was identified in approximately one third of tumors. No significant associations were observed between ERα, ERβ or AR expression and overall, cancer-specific or recurrence-free survival. This is the first study to investigate sex hormone receptor expression in EAC patients by tumor location and there was a suggestion that ERβ expression was associated with a reduction in the risk of all-cause and cancer-specific death in patients with GEJ cancer, albeit findings did not reach statistical significance.

Previous research into sex hormone receptor expression in EAC has been sparse and contradictory (see Table 4). Similar to our study, earlier studies have identified positive ERβ expression [24–26, 28] and none or limited ERα expression [24, 25] in EAC. Additionally, in a small study of 33 patients, Liu *et al*. [26] reported that ERβ subtype isoforms (ERβ1, ERβ2, ERβ3 and ERβ5) were overexpressed in EAC compared to precursor lesions. Similar to our study, Al-Kyatt *et al*. [24] found limited ERα receptor expression (*n* = 1 case, defined as a H-score of 10), while moderate ERβ receptor expression was detected (*n* = 14 cases (40%), defined as a H-score of 30) [24]. Although the authors did not separate findings by histological subtype, the majority of the cohort (76%) had EAC. The authors also used quantitative reverse transcription polymerase chain reaction to evaluate ER tumor expression and reported that ERβ expression (ESR1, based on mRNA levels) was correlated with a shorter 1-year disease-specific survival (*p* = 0.05), suggesting that ERβ may be a marker of poor biological behavior in EAC [24]. However, most likely due to small numbers (*n* = 28 EAC patients), a formal survival analysis was not conducted therefore limiting comparison with our study.

**Table 4:** Previous studies of ERα, ERβ and AR expression and survival in esophageal adenocarcinoma cohorts

Study (year)	Location	Hormone receptor	Number of patients		Antibody (dilution)	Positive expression cut-off value (range)	Mortality	HR (95% CI)	*p*
			Positive expression	Negative expression					
Al-Kyatt (2018)^a^	UK	ERα	1	33	Mouse (1:40)	H-score ≥ 10	CS	NR	0.05^b^
		ERβ	14	20			CS	NR	0.02^b^
Kalayarasan (2008)^c^	India	ERα	0	15	Mouse (Prediluted)	Quick score ≥ 1 (0–7)^d^	NR	NR	NR
		ERβ	15	0	Mouse (1:50)				
Liu (2004)	USA	ERβ-1	23	4	NR	≥1%^e^	NR	NR	NR
		ERβ-2	22	5					
		ERβ-3	27	0					
		ERβ-5	27	0					
Akgun (2002)	USA	ERβ	23	0	Rabbit (1:500)	≥1% staining in cells	NR	NR	NR
Awan (2007)	UK	AR	13	5	Mouse (1:50)	Any focal/diffuse staining^f^	AC	NR	NS
Tiffin (2003)	UK	ER	8	12	NR	Any mild, moderate or heavy staining^g^	NR	NR	NR
		AR	1	19					
Tihan (2001)	USA	AR	5	6	Mouse (1:200)	Any focal/diffuse staining^h^	AC	NR	NS

Abbreviations: ER = estrogen receptor, AR = androgen receptor, NR = Not reported; NS = Non-significant; AC = All-cause; CS = Cancer-specific, α = alpha; β = beta

^a^Includes esophageal adenocarcinoma (*n* = 28) and esophageal squamous cell carcinoma (6) patients.

^b^Survival correlated with ERα (ESR1) and ERβ (ESR2) expression quantified using quantitative reverse transcription polymerase chain reaction.

^c^Endoscopic biopsy specimens used to determine sex hormone receptor expression.

^d^Receptor expression reported as ‘0 = no staining’; ‘1 = weak intensity (only visible at high magnification)’; ‘2 = moderate intensity (readily visible at low magnification)’; ‘3 = strong (strikingly positive even at low power. magnification)’; added to score for % of cells stained (0 = 0%; 1 = 1–25%; 2 = 26–50%; 3 = 51–75%; 4 = 76–100%). Mean quick score was 6.6 for ERβ.

^e^Receptor expression reported as % of cells stained (0 = 0%; 1 = 1% to 10%; 2 = 11% to 25%; 3 = 26% to 50%; 4 = 51% to 75%; 5 ≥75%).

^f^Receptor expression reported as ‘no staining’; ‘focal positivity’ or ‘diffuse positivity’.

^g^Receptor expression reported as ‘absent’; ‘mild staining’; ‘moderate staining’ or ‘heavy staining’.

^h^Receptor expression reported as ‘no staining’; ‘focal positivity’ or ‘diffuse positivity’.

In a small study of 31 EAC patients with available esophagectomy specimens, Akgun and colleagues [28] reported expression of ERβ in Barrett's metaplasia negative for dysplasia, low-grade dysplasia, high-grade dysplasia and adenocarcinoma and observed a trend of increased expression as esophageal lesions progressed. Similarly, other studies have noted higher expression of ERβ in EAC compared to Barrett's esophagus [26] and normal esophageal mucosa [25], and poorer differentiation in EAC tumors that express ERβ [25, 28]. We did not find any significant differences in tumor grade according to sex hormone receptor expression. Some [30, 29] but not all previous studies [27] have reported weak-to moderate positive expression for AR in EAC with no differences in survival noted by expression status [30, 29] but sample sizes have been small (e.g. less than 20 EAC patients). In a small study of 11 EAC patients, Tihan *et al*. [30] noted that patients with AR-negative tumors included a larger proportion of longer-term survivors compared to patients with AR-positive tumors but no statistically significant difference in survival was apparent and no formal survival analysis was conducted. Variation in terms of sample sizes, selected patient groups, methods used to quantify receptor expression, as well as a lack of adjustment for important confounders in previous studies make direct comparisons with our study difficult.

In our study, positive expression of ERβ was predominantly limited to males, which could be suggestive of a potential biological role for estrogen and/or ERβ receptor in EAC development. Within the minority of patients whose tumors expressed ERα and AR, the majority of expression was also detected in male EAC patients. Preclinical data have shown that estrogen and selective estrogen receptor modulator treatment decreases tumor growth, proliferation and increase apoptosis in EAC and Barrett's esophagus cell lines [13, 14] and similar effects have been reported *in vitro* and *in vivo* for gastric [31, 32] and colorectal cancer [33] and esophageal squamous cell carcinoma (OSSC) [20, 34]. Interestingly, ERβ has been correlated with improved survival in non-small cell lung adenocarcinoma, particularly in men [35], although findings from other studies have been mixed [36, 37] and a recent pooled analyses found no association between ERβ expression and survival in NSCLC patients [38].

A possible mechanism of estrogen ‘protection’ might be mediated through estrogen receptors however, the role of activation of these receptors in re-epithelialisation following injury, and in the development of precursor states and EAC remains unclear. AR has been shown to be an important mediator of inflammatory signals in esophageal cancer progression [20] and overexpression of AR *in vivo* has been shown to promote cell migration, invasion and proliferation in esophageal cancer [13, 20]. Although risk estimates for EAC survival in our study suggested potential inverse associations for ERβ and positive associations for AR expression, our results were not statistically significant. Expression of sex hormone receptors, in particular ERβ, in EAC and their relationship with EAC development and progression therefore merits further investigation in larger population-based cohorts. Moreover, studies should include sufficient numbers of females to enable more meaningful sex-specific comparisons.

In our study, sensitivity analysis suggested that ERβ expression was associated with more marked reductions in all-cause and cancer-specific mortality in patients diagnosed with GEJ cancer. Although not as marked as EAC, gastric cardia adenocarcinomas, located close to the GEJ, show a similar sex disparity in incidence compared to EAC [10, 11] and proximal gastric cancers share similar aetiology and patterns of incidence with EAC [39]. Increased ERβ expression has been demonstrated in gastric cancer tissue compared to normal tissue [40, 41] and its presence has been associated with poorer prognosis [42, 43] however, other studies have shown no such association [40, 44]. No study has specifically investigated ERβ expression in cancer tissues of the proximal stomach. Epidemiological studies of markers of endogenous and exogenous estrogen exposures have suggested protective associations for gastric cancer [45], which are possibly more marked for gastric cardia adenocarcinomas [46], and preclinical evidence shows an increase in apoptosis and an inhibition of cell migration with estrogen administration in gastric cancer cell lines [32, 47]. A more marked prognostic benefit for ER receptor expression in GEJ tumors is therefore plausible but findings require replication in other large EAC cohorts. We did not have information on sex hormone receptor isoforms, which limited our ability to identify overexpression events directly linked to clinical EAC outcomes. Future studies should aim to investigate the complex interplay between wild-type and variant forms of sex hormone receptor expression in EAC and their relationship with prognosis. Moreover, considering the potential for inter- and intra-nuclear receptor crosstalk within ER, studies that examine alternative gene splicing via transcriptomic analysis of EAC tumour specimens both before and after chemotherapy may be warranted. Despite being the largest study conducted to examine the association between ERβ expression and EAC progression, caution is required in the interpretation of these results for GEJ patients as analysis was secondary in nature and based on reduced number of patients.

Our study had a number of strengths and limitations. This is the first population-representative study to investigate sex hormone receptor expression in EAC tissue and their association with prognostic outcomes. Although we restricted our cohort to patients who underwent neoadjuvant chemotherapy prior to surgical resection, neoadjuvant therapy is now an integral component of EAC management [48] and we included all patients who underwent these treatments in Northern Ireland during the study period. Although progesterone receptor expression has been shown to be absent in both normal epithelial mucosa and oesophageal tumours [24], we did not investigate its expression, or expression of membrane bound G-protein coupled receptors (e.g. G protein-coupled estrogen receptor 1; GPER or GPR30). Future studies should explore the role of these additional receptors in OAC progression. Our study benefitted from a long follow-up period of (up to 9 years) and we had detailed clinico-pathological information, which allowed adjustment for confounders. Two observers (including a trained pathologist) independently scored all tumor cores and were blinded to clinical data. We utilised EAC tissue treated with chemotherapy, however it is unclear how treatment influences expression of sex hormone receptors. We used triplicate tumor cores to generate H-scores, thus limiting the potential for sampling bias. Insufficient tissue in diagnostic biopsies prohibited investigation of sex hormone receptor expression in EAC tissue prior to chemotherapy administration; however, pre-treatment endoscopic biopsies may be subject to sampling error. Finally, although our study is the largest to date to examine sex hormone receptor expression and EAC outcomes, expression of ERα and AR was observed in only a minority of patients and small case numbers limited sensitivity and sub-group analyses (e.g. by sex).

## MATERIALS AND METHODS

### Study cohort

We included all EAC patients who underwent neo-adjuvant chemotherapy prior to surgical resection in Northern Ireland between 2004 and 2012. All patients received cisplatin-based neo-adjuvant chemotherapy at the Northern Ireland Cancer Centre followed by surgical resection at one of four regional surgical centres. EAC resection specimens were accessed through the Northern Ireland Biobank (NIB) (study number NIB15-0176) which has ethical approval for the collection and storage of tumor tissue to support translational research [49]. To be included, patients had to have non-metastatic disease, have at least one available TMA tissue core with sufficient tumor for immunohistochemical scoring and have available clinico-pathological data. This study is in accordance with the Reporting Recommendations for Tumor Marker Prognostic Studies (REMARK) guidelines [50].

### Clinico-pathological variables and follow-up

Clinical data were retrieved from a medical note review within the Northern Ireland Cancer Centre and pathology reports were reviewed for information on TNM stage, tumor grade, primary tumor site, positron emission tomography (PET) response, lymphovascular invasion and circumferential margin involvement. EAC tumors were classified according to location using the Siewert classification [51]. Pathological staging was defined according to International Union Against Cancer (UICC) TNM staging, 7th edition. Information on study outcomes were retrieved from the Northern Ireland Cancer Centre and included overall survival (time to death from any cause), cancer-specific survival (time to death from EAC) and recurrence-free survival (time to clinical or pathological recurrence or death). Patients were followed from the date of EAC diagnosis until recurrence, death or end of study follow-up (31st December 2014).

### Tumor molecular analysis

#### Tissue microarray construction and immunohistochemistry

Tissue microarrays (TMAs) were created within the Northern Ireland Molecular Pathology Laboratory (NI-MPL) at Queen's University Belfast. A detailed description of TMA construction methods within the Northern Ireland Biobank are described elsewhere [52]. Immunohistochemical analysis was also conducted within the NI-MPL, where all markers were validated to investigate their association with other tumor sites (e.g. breast, prostate). A 3-μm thick section was deparafinized and endogenous peroxidase activity quenched with 0.3% hydrogen peroxide prior to slide labelling and staining using the Ventana Discovery XT^®^ automated immunostainer (Ventana Medical Systems Inc, Tuscon, AZ) or the Bond Automated IHC/ISH Stainer (Leica Biosystems, Milton Keynes, UK). Slides were stained with rabbit (ERα) or mouse (AR, ERβ) monoclonal antibodies specific to the relevant sex hormone receptor. Antibody commercial suppliers included Ventana (cat no. 790-4324) for ERα, Dako (cat no. M7292) for ERβ and Abcam (cat no. ab9474) for AR. Cell signalling-clone numbers (and dilutions) were as follows; ERα: SP1 (RTU dilution), ERβ: PPG5/10 (dilution 1 in 100) and AR: AR441 (1 in 200 dilution).

### Biomarker scoring and assessment

Individual biomarker expression was assessed by two independent observers (JT & ÚMcM) using an online image viewer PathXL, following training and guidance from an expert gastrointestinal pathologist (DMcM). Both observers were blinded to clinical data. Nuclear, cytoplasmic or cell membrane staining was considered and we quantified sex hormone receptor expression by calculating H-scores that incorporated staining intensity and frequency, with consensus agreement of discordant results. Scoring was based on intensity (0 = no staining, 1 = weak, 2 = moderate and 3 = strong staining observed) and percentage of tumor cells staining positive. These two values were multiplied to give a H-score between 1 and 300 and the maximum H-score from the three cores was used for analysis. A dichotomous classification was used to categorise maximum H-scores into positive (any) or negative expression.

### Statistical analysis

Differences between clinico-pathological characteristics according to ERα, ERβ and AR expression were calculated using chi-squared tests. Associations between sex hormone receptor expression (positive versus negative) and recurrence-free, cancer-specific and overall survival were investigated using Cox proportional hazards regression producing unadjusted and adjusted hazard ratios (HR) and 95% confidence intervals (CIs). All analyses were adjusted for age at diagnosis, sex, pathological nodal stage, grade, primary site (lower third or gastro-esophageal junction (GEJ)), lymphovascular invasion, circumferential margin involvement, PET response and smoking. An unknown category was included for missing values for smoking and PET response. Sensitivity analysis was conducted restricting the cohort to patients diagnosed with cancers of the GEJ. Due to the small number of females included, we were unable to conduct sub-group analysis by sex. All statistical analysis was performed using STATA 14 (StataCorp LP, College Station, TX, USA).

## CONCLUSIONS

In a population-based cohort of EAC patients who underwent neo-adjuvant chemotherapy prior to surgical resection, we found little evidence of ERα or AR expression in EAC. Expression of ERβ was identified in one third of tumors, and although no significant associations were observed for the whole cohort, there was suggestive evidence that ERβ expression was associated with improved survival in patients with GEJ cancer. Considering that surgery alone is not a curative option for all stages of EAC and further therapeutic modalities are urgently required, further investigation is warranted to determine if the ER system could be a potential prognostic biomarker in EAC.

## Abbreviations

EAC: esophageal adenocarcinoma
ER: estrogen receptor
AR: androgen receptor
FFPE: formalin-fixed paraffin embedded
GEJ: gastro-esophageal junctional
yp: pathologic stage classification determined after preoperative therapy
